# An annotated checklist of millipede fauna from Slovakia, with ecological and biogeographic characteristics

**DOI:** 10.3897/BDJ.9.e71495

**Published:** 2021-09-09

**Authors:** Beáta Haľková, Martina Drabová, Andrej Mock

**Affiliations:** 1 Pavol Jozef Safarik University, Faculty of Science, Košice, Slovakia Pavol Jozef Safarik University, Faculty of Science Košice Slovakia

**Keywords:** Diplopoda, ecological classification, distributional pattern, faunal list, new records

## Abstract

**Background:**

Two decades have passed since the publishing of the last checklist of the millipedes of Slovakia. During this time, several new faunistic records have been added and taxonomic revisions have occurred. The present updated checklist summarises data on all millipede species recorded in Slovakia, including altogether 93 species.

**New information:**

For each species, general habitat characteristics, ecological classification and distributional pattern are provided. Ecological classification is presented for the first time for the millipede species occurring in Slovakia and is proposed as a tool for ecological studies and for the nature protection purposes. Special remarks are given to the species newly found for Slovakia, *Geoglomerissubterranea* Verhoeff, 1908, *Brachyiuluslusitanus* Verhoeff, 1898, *Cylindroiulusbritannicus* (Verhoeff, 1891), *C.parisiorum* (Brölemann & Verhoeff, 1896) and *Polydesmusburzenlandicus* Verhoeff, 1925, as well as to *C.arborum* Verhoeff, 1928, the species newly confirmed for Slovakia after more than 70 years.

## Introduction

Until 1993, Slovakia was part of several political units (Hungarian, Austro-Hungarian Monarchy, Czechoslovakia, Slovak Republic, Hungary); therefore, historical data on Slovak millipede fauna are not easy to find and there has been a lot of geographical confusion in trying to locate old published records. The studies on the millipedes in Slovakia have a long history, with the first papers published at the end of the 19^th^ century (Nowicki 1867, Nowicki 1869, Nowicki 1870, Nowicki 1871, Karliński 1883a, Karliński 1883b, Latzel 1882, Latzel 1884, Chyzer 1886, Daday 1889, Daday 1896, Petricskó 1891, Petricskó 1892, Malesevics 1892, Attems 1895, Attems 1899, Verhoeff 1899a, Verhoeff 1899b). One of the most important authors working at that time in Slovakia was Ödön Tömösváry. He wanted to prepare a monograph on the myriapods of the Kingdom of Hungary, but died young and his findings were at least excerpted in the papers of Chyzer (1886), Daday (1889) and Daday (1896).

In the pre-war and interwar periods, K. W. Verhoeff, the phenomenal German zoologist, expanded the knowledge of the millipedes of our country. In addition, important information about the millipede fauna of Slovakia is included in historical monographs published by Attems (1926), Verhoeff (1928), Verhoeff (1932), Verhoeff (1937), Ortvay (1902) and Schubart (1934). A significant contribution to the knowledge of Slovak millipedes during the first half of the 20^th^ century was done by other authors, including Jawlowski (Jawlowski 1930, Jawlowski 1938), Jermy (1942) and Loksa (Loksa 1954, Loksa 1957, Loksa 1962, Loksa 1968). In the second half of the 20^th^ century, the most extensive research on millipedes in Slovakia was performed by Ján Gulička (1925–2009) (Gulička 1952, Gulička 1954, Gulička 1955, Gulička 1956a, Gulička 1956b, Gulička 1957, Gulička 1960b, Gulička 1965, Gulička 1985, Gulička 1986). Gulička's unpublished compendia (Gulička 1951, Gulička 1960a), which we have today and can work with, were not taken into account in the last published checklist (Mock 2001a). This last checklist included all the available information at that time and involved information for about 70 species reported for the whole territory of Slovakia. A new chapter of millipede research began at the end of the 20^th^ century, conducted by several researchers, for example, Tajovský (1997), Mock (Mock 1998, Mock 1999a, Mock 1999b) and Stašiov (Stašiov 1997, Stašiov 1998). Several studies on Slovak millipede fauna were published after Gulička’s death (Gulička 2016, *Gulička et al. 2014, Gulička and Košel 2016*), based on material and manuscripts from his estate. Besides this, Gulička's unpublished compendia (Gulička 1951, Gulička 1960a), which we have today available and data listed there can be actually re-evaluated and taken into consideration, represent other sources for more complex overview of the millipede fauna of Slovakia.

Since 2001, the number of species has changed significantly, thus warranting a new update. The aim of this report is to update the list of millipede species and their distribution in Slovakia, to summarise published, as well as unpublished data and records, respecting recent taxonomic changes and to provide a useful tool in the form of ecological and geographical classification of species for other studies.

## Materials and methods

The classification for Diplopoda used for this checklist follows the systematic arrangement by Shear (2011), while the nomenclature follows Kime and Enghoff (2011), Kime and Enghoff (2013), Kime and Enghoff (2017). We verified the system and nomenclature using MilliBase (Sierwald and Spelda 2021); however, we are reticent towards some proposals, for example, dividing the genera *Brachydesmus* and *Polydesmus* into other genera.

We do not include the subspecific categories as subspecies, nor all the relevant literature since that information has been included by the author of the last published checklist (Mock 2001a). References are listed at the species where the first records for Slovakia were documented for the first time after the year 2000. In addition, habitat evaluation for each species is included, applied specifically to the area of Slovakia, following adapted categorisation proposed by Tuf and Tufová (2008) and marked by a letter in caps:

- **Relic species (R)** - stenotopic species inhabiting exclusively undisturbed habitats with low impact of human activities, for example, the nature-closest forests, remnants of steppes, caves, stony debris, mountain habitats.
- **Adaptable species (A)** - species inhabiting nature-close habitats (various types of forests or meadows), able to penetrate artificial and man-made habitats (parks, abandoned gardens, graveyards, greenhouses etc.).
- **Eurytopic species (E)** - species with the widest ecological valence, frequently found in forested and non-forested biotopes and in man-made plots (fields, brownfields etc.).
- **Synanthropic species (S)** - species inhabiting a wide spectrum of man-made habitats, missing in natural biotopes, species significantly synanthropic, although occasionally found in natural, mainly altered, sites.
- **Exotic species (X)** – this category includes mostly tropical species, non-native or introduced species, found exclusively in hothouses.

Ecological classification of each species (Kime and Enghoff 2011, Kime and Enghoff 2017) is provided in Notes, marked by lowercase letters:

- **(c)** - corticolous
- **(e)** - epigeic
- **(ed)** - edaphic
- **(h)** - hygrophilous
- **(m)** - mountainous
- **(n)** - nidicolous
- **(tb)** - troglobitic
- **(tp)** - troglophilous
- **(x)** - xerophilous

We would like to note that we did not adopt this habitat evaluation scheme mechanically, but we evaluate each species, based on the knowledge about its ecology in Slovakia. Question mark **(?)** next to the classification refers to problematic data on the occurrence of the species.

In addition, a general geographical category that outlines the distribution of each species is included in the list (Kime and Enghoff 2011, Kime and Enghoff 2017).

## Data resources

Records from Slovakia compiled from the published literature and additional collected material are summarised. The actually sampled material is in the collection at the workplace of the authors' team. Part of the data was obtained by preliminary revision of the collection by J. Gulička, including his unpublished compendia. A large portion of Gulička's complete written legacy is lost. However, his views on the taxonomy, biogeography and ecology of individual species can be found in Gulička's additionally handwritten notes in the monographs of other authors (e.g. Verhoeff 1928, Schubart 1934 etc.).

## Checklists

### Updated list of the millipede fauna of Slovakia

#### Polyxenida

51B5879F-5FC7-5A53-8809-BE8BA4E6EFD4

#### Polyxenidae

1474AEEA-6B12-5591-ACB8-91EDF280965C

#### Polyxenus lagurus

(Linnaeus, 1758)

828426A7-1F69-533A-8D81-0CB37BEC7213

##### Distribution

West Palearctic and Nearctic

##### Notes

A, c, n

#### Glomerida

BFC7F491-9FAA-5D78-ADF4-E7B530A4DD4E

##### Notes

The order Glomerida, according to published data, is represented by three genera in Slovakia – *Trachysphaera, Glomeris* and *Geoglomeris* (Lang 1954, Kime and Enghoff 2011, Kime and Enghoff 2013, Kocourek et al. 2017). Genus *Glomeris* is rather problematic, as it represents a complex of species, characterised by inconsistency and unreliable determination information in the old literature. *G.tetrasticha*, *G.hexasticha* and *G.pustulata* represent well-confirmed species, occurring in the territory of Slovakia. Only one old record of *G.klugii* from Slovakia exists. In the area of Slovakia, some populations of the species of the genus *Glomeris* are characterised by a remarkable variety of colouration, which could indicate the ongoing speciation processes.

#### Glomeridae

EBD87C29-C98C-5A2D-8A64-999815A34F72

#### Glomeris hexasticha

Brandt, 1833

A2511E1B-A91D-5850-ABB7-F2C07503C54C

##### Distribution

South, Central and East European

##### Notes

A, e, x

#### Glomeris klugii

Brandt, 1833

61A6C9BA-E61A-533A-8F6C-4DA9CA387815

##### Distribution

West and South European

##### Notes

A, e, ?

The species (known under synonyms *G.conspersa* and *G.undulata* in older literature) is characterised by a south-western occurrence in Europe (Kime and Enghoff 2011). It has been confirmed in most of the neighbouring countries (Poland, Czech Republic, Austria and Hungary). For Slovakia, only one record of this species has been recently discovered, in the millipede collection of Czech arachnologist František Miller (1902–1983), housed in the National Museum, Prague (Dolejš and Kocourek 2020). One female specimen of *G.klugii* is included in the collection, labelled with April 1930, Turčianske Teplice District, Žilina Region. Based on this information, a series of sampling from this area has been conducted, but unsuccessfully. Therefore, a mistake in localisation made by collector is highly possible.

**Reference**: Dolejš and Kocourek (2020)

#### Glomeris mnischechi

Nowicki, 1870

694103EA-A3AF-533E-A057-1E6D5868BA6A

##### Distribution

West Carpathian

##### Notes

R, e

*Glomerismnischechi* is considered the only endemic species of the order Glomerida north of the Alps. According to Kime and Enghoff (2011), the species inhabits mountainous biotopes of Slovakia (Belianske Tatras Mts., Pieniny Mts.) and Poland. However, literature contains many controversial data about diagnostic characteristics, as well as ecological demands (Mock 2001a, Kravcová and Mock 2014). Nowicki originally offered two descriptions of *G.mnischechi* (Nowicki 1870, Nowicki 1871), without any illustration. In addition, the author used different transcription for the species names in both cases, *Glomerismnischechi* (Nowicki 1870) and *Glomerismniszechii* (Nowicki 1871). After this confusion, the species was described several times in the old literature, under various synonyms (Latzel 1884, Gulička 1951, Gulička 1960a, Dziadosz 1966). Other authors considered the species to be subspecies of *G.hexasticha*, due to the striking similarity of both species (e.g. Jawlowski 1938). Although the taxonomic status of the species remains unresolved, recent molecular analyses of several representatives of *G.hexasticha* from the type locality of *G.mnischechi* suggest the presence of several separate species; therefore, the existence of *G.mnischechi* and its occurrence in Slovakia cannot be ruled out.

#### Glomeris pustulata

(Fabricius, 1781)

8EAAF47F-F7BE-5D57-AD24-C8CA0B7A8FC5

##### Distribution

South and Central European

##### Notes

A, e, c

#### Glomeris tetrasticha

Brandt, 1833

D64F6E1F-0B9A-5880-9006-DBADB01E7E7C

##### Distribution

Central and East European

##### Notes

A, e

Some authors mentioned the occurrence of *Glomerisconnexa* C. L. Koch, 1847 in Slovakia. This species is characteristic for its south-western European distribution (Kime and Enghoff 2011). In the literature from the last century, there is a lot of data on the findings and its occurrence. However, it should be noted that, in most cases, under this name, there were referred other species, especially *G.tetrasticha* (Kime and Enghoff 2011, Kocourek et al. 2017). From Slovakia, no recent reliable records of its occurrence are available; therefore, the older data on this species name are preliminarily all included under *G.tetrasticha*. However, due to the findings from the Czech Republic, very close to the border with Slovakia, the occurrence of this species cannot be ruled out.

#### Geoglomeris subterranea

Verhoeff, 1908

485B724B-7148-51A6-9F8A-F595371C7499

##### Distribution

West and Central European

##### Notes

R, ed, tp, h

This species was described by Verhoeff (1908) from the neighbourhood of Dresden, Germany, where two females were found on limestone near a brook. In his monograph on the millipede fauna of Czechoslovakia, Lang (1954) presented the finding of *G.subterranea* in the only Slovak locality from the vicinity of the Bratislava City (Malé Karpaty Mts.). However, he did not comment on the finding and attached only a picture taken from other literature (Schubart 1934). Since the cited monograph contains many ambiguities and unreliable data, this information is considered doubtful (Gulička 1986, Mock 2001a). In the area of Slovakia, it was found only recently (Haľková et al., unpublished), repeatedly, in karst springs and wetlands on karst bedrock. Detailed morphological study (including SEM), supported by molecular analysis, confirmed the identity of *G.subterranea*, without any apparent morphological adaptations to aquatic and semi-aquatic habitat. The possibility that this species is not strictly limited to the terrestrial environment has already been suggested by Noll (1939), although his findings were completely forgotten in recent literature. Noll mentioned the presence of *Geoglomerisjurassica* (a younger synonym of *G.subterranea*) in the water of three wells in Northern Bavaria, Germany. The author explained its occurrence as random, presuming the animals entered the well through crevices in the wall.

#### Trachysphaera acutula

(Latzel, 1884)

E2F129A8-8841-5B7A-8884-F673C64DA7E8

##### Distribution

Carpathian

##### Notes

R, ed, tp, h

#### Trachysphaera costata

(Waga, 1857)

B05A6FD8-BBE1-5C39-87F4-27EE0DDCB1E5

##### Distribution

East and Central European

##### Notes

R, ed, m

#### Trachysphaera gibbula

(Latzel, 1884)

C8D3F0D0-4F6A-570C-A64D-2544106EAECD

##### Distribution

Alpine and West Carpathian

##### Notes

R, ed

#### Polyzoniida

0D81710B-775D-5A5F-B443-9D6023099906

#### Polyzoniidae

621915C8-EAFC-5396-9714-FE20D7462F70

#### Polyzonium eburneum

Verhoeff, 1907

F24C4010-6F24-56CD-9527-A5CFB0D89E06

##### Distribution

East Alpine and West Carpathian

##### Notes

R, e

#### Polyzonium germanicum

Brandt, 1837

0FF2874E-FB0B-579D-AE75-912804D3A386

##### Distribution

European

##### Notes

A, e, h

#### Polyzonium transsilvanicum

Verhoeff, 1898

6E9952B6-3AB8-57ED-927F-53563C7C8C6C

##### Distribution

East Carpathian

##### Notes

R, h

#### Julida

85AF59DC-241D-5259-BB9E-A2B5FD794658

#### Blaniulidae

EFAA8300-886A-5590-B1A3-EAECD325EF46

#### Archiboreoiulus pallidus

(Brade-Birks, 1920)

3481208A-BB4B-5F5A-8FB5-5089E9D8B446

##### Distribution

West, North, Central and East European

##### Notes

R, ed, tp

**Reference**: Mock et al. (2015)

#### Blaniulus guttulatus

(Fabricius, 1798)

B89DF54C-253C-5767-887F-BF4C2CF3B1A6

##### Distribution

European

##### Notes

S, ed

#### Cibiniulus slovacus

Antić, Mock & Enghoff, 2015

6BD50117-1D65-52DF-B16F-788C6FE3D833

##### Distribution

West Carpathian

##### Notes

R, ed, tp

#### Choneiulus palmatus

(Němec, 1895)

58F43795-2A1E-5F3A-917B-9BBF992DCF83

##### Distribution

European

##### Notes

S, ed

**Reference**: Mock (2001b)

#### Nopoiulus kochii

(Gervais, 1847)

82D904AB-2C93-5895-9895-696840D9AD3B

##### Distribution

Euro-Caucasian

##### Notes

S, ed

#### Proteroiulus fuscus

(Am Stein, 1857)

90CD54F7-E609-5EEA-A47D-CA7C9BFB183F

##### Distribution

European

##### Notes

E, c, n

#### Nemasomatidae

17D6B204-1121-5909-8A04-30BB23FCB962

#### Nemasoma varicorne

C. L. Koch, 1847

E2AE1783-D0ED-5389-99E3-EA3FDCEA4048

##### Distribution

European

##### Notes

R, c, n

#### Julidae

A0FD6BA1-3FBD-5217-908B-7229ADD0FA3B

#### Brachyiulus bagnalli

(Brolemann, 1924)

8A120E9F-8E37-5E28-A5AE-266719F586D5

##### Distribution

South and Central European

##### Notes

E, e, n, x

#### Brachyiulus lusitanus

Verhoeff, 1898

DA264670-66AC-5322-B965-09E7A9B62558

##### Materials

- **Type status:**Other material. **Occurrence:** individualCount: 1; sex: male; lifeStage: adult; **Taxon:** scientificName: *Brachyiuluslusitanus* Verhoeff, 1898; class: Diplopoda; family: Julidae; genus: Brachyiulus; specificEpithet: *lusitanus*; taxonRank: species; **Location:** country: Slovakia; locality: Košice Basin, Košice – a city park (Mestský park) at the railway station; verbatimElevation: 210 m a.s.l.; locationRemarks: leaf litter (*Acer platanoides, Aesculus hippocastaneum*); verbatimCoordinates: 48°43'20.4"N 21°15'52.5"E; **Identification:** identifiedBy: A. Mock; **Event:** eventDate: 18-12-2018; fieldNotes: co-existing with *Nopoiuluskochii, Cylindroiulusparisiorum, Ophyiuluspilosus*; **Record Level:** basisOfRecord: PreservedSpecimen
- **Type status:**Other material. **Occurrence:** individualCount: 2; sex: female; lifeStage: adult; **Taxon:** scientificName: *Brachyiuluslusitanus* Verhoeff, 1898; class: Diplopoda; family: Julidae; genus: Brachyiulus; specificEpithet: *lusitanus*; taxonRank: species; **Location:** county: Slovakia; locality: Košice Basin, Košice – a city park (Mestský park) at the railway station; verbatimElevation: 210 m a.s.l.; locationRemarks: leaf litter (*Acer platanoides, Aesculus hippocastaneum*); verbatimCoordinates: 48°43'20.4"N 21°15'52.5"E; **Identification:** identifiedBy: A. Mock; **Event:** eventDate: 18-12-2018; fieldNotes: co-existing with *Nopoiuluskochii, Cylindroiulusparisiorum, Ophyiuluspilosus*; **Record Level:** basisOfRecord: PreservedSpecimen

##### Distribution

European

##### Notes

A, e

According to Kime and Enghoff (2017), the species is distributed in Western and Central Europe and Balkans, but also Algeria, Egypt and Iran. It was introduced into Australia and North America. The species can be found in forests, in addition to open land, meadows, cornfields and vineyards. It has been recorded under stone debris, as well as city parks and heated greenhouses.

#### Cylindroiulus boleti

(C. L.Koch, 1847)

4B2E539A-C953-5C56-A2A3-B021375BA5F8

##### Distribution

South and Central European

##### Notes

R, e

#### Cylindroiulus arborum

Verhoeff 1928

B89DFBDB-1AE3-5FF3-9F8F-FC29FCCBABC9

##### Materials

- **Type status:**Other material. **Occurrence:** individualCount: 1; sex: male; lifeStage: adult; **Taxon:** scientificName: *Cylindroiulusarborum* Verhoeff, 1928; class: Diplopoda; family: Julidae; genus: Cylindroiulus; specificEpithet: *arborum*; taxonRank: species; **Location:** country: Slovakia; locality: Burda Mts.; verbatimElevation: 190 m a.s.l.; locationRemarks: forest (*Quercus* spp., *Carpinusbetulus*, *Fraxinusexcelsior*), decomposed wood from an oak-tree cavity; verbatimCoordinates: 47°50'39.84"N 18°49'16.92"E; **Identification:** identifiedBy: A. Mock; **Event:** eventDate: 2017-06-16; fieldNotes: co-existing with *Proteroiulusfuscus* and juveniles of *Haaseaflavescens*; **Record Level:** basisOfRecord: PreservedSpecimen
- **Type status:**Other material. **Occurrence:** individualCount: 1; sex: female; lifeStage: adult; **Taxon:** scientificName: *Cylindroiulusarborum* Verhoeff, 1928; class: Diplopoda; family: Julidae; genus: Cylindroiulus; specificEpithet: *arborum*; taxonRank: species; **Location:** country: Slovakia; locality: Burda Mts.; verbatimElevation: 190 m a.s.l.; locationRemarks: forest (*Quercus* spp., *Carpinusbetulus*, *Fraxinusexcelsior*), decomposed wood from an oak-tree cavity; verbatimCoordinates: 47°50'39.84"N 18°49'16.92"E; **Identification:** identifiedBy: A. Mock; **Event:** eventDate: 2017-06-16; fieldNotes: co-existing with *Proteroiulusfuscus* and juveniles of *Haaseaflavescens*; **Record Level:** basisOfRecord: PreservedSpecimen
- **Type status:**Other material. **Occurrence:** individualCount: 16; lifeStage: juveniles; **Taxon:** scientificName: *Cylindroiulusarborum* Verhoeff, 1928; class: Diplopoda; family: Julidae; genus: Cylindroiulus; specificEpithet: *arborum*; taxonRank: species; **Location:** country: Slovakia; locality: Burda Mts.; verbatimElevation: 190 m a.s.l.; locationRemarks: forest (*Quercus* spp., *Carpinusbetulus*, *Fraxinusexcelsior*), decomposed wood from an oak-tree cavity; verbatimCoordinates: 47°50'39.84"N 18°49'16.92"E; **Identification:** identifiedBy: A. Mock; **Event:** eventDate: 2017-06-16; fieldNotes: co-existing with *Proteroiulusfuscus* and juveniles of *Haaseaflavescens*; **Record Level:** basisOfRecord: PreservedSpecimen

##### Distribution

Central and East European

##### Notes

R, e

Mainly lowland species with Central and East European distribution (Kime and Enghoff 2017). The species usually prefers forest habitats, found mostly in deadwood and leaf litter, although several records are from hothouses and other artificial habitats. New record after more than 70 years in Slovakia (see Dudich 1958, Mock 2001a).

#### Cylindroiulus britannicus

(Verhoeff, 1891)

00B4D62F-CAB4-5AC1-A501-206AD0322D4C

##### Materials

- **Type status:**Other material. **Occurrence:** individualCount: 5; sex: male; lifeStage: adult; **Taxon:** scientificName: *Cylindroiulusbritannicus* (Verhoeff, 1891); class: Diplopoda; family: Julidae; genus: Cylindroiulus; specificEpithet: *britannicus*; taxonRank: species; **Location:** country: Slovakia; locality: Košice Basin, Košice, Public cemetery; verbatimElevation: 225 m a.s.l.; locationRemarks: leaf litter (*Tilia cordata, Fraxinusexcelsior, Acer platanoides*); verbatimCoordinates: 48°41'42.420"N 21° 15'35.130"E; **Identification:** identifiedBy: A. Mock; **Event:** eventDate: 2020-05-26; fieldNotes: co-existing with *Blaniulusguttulatus*, *Choneiuluspalmatus, Cylindroiulus caeruleoncictus, Ophyiuluspilosus*; **Record Level:** basisOfRecord: PreservedSpecimen

##### Distribution

Cosmopolitan

##### Notes

S, c, e

Species with European distribution, in Britain and Ireland found beneath the bark of dead deciduous tree trunks and stumps (Blower 1985). It appears to be predominantly or entirely synanthropic in most other countries, occurring primarily in cities and cultivated areas (Kime and Enghoff 2017). The same applies to the first findings of the species in Slovakia, where it was found in city parks.

#### Cylindroiulus burzenlandicus

Verhoeff, 1907

87350C1D-256A-5255-80AB-B78FB5330452

##### Distribution

Carpathian

##### Notes

R, e, m

#### Cylindroiulus caeruleocinctus

(Wood, 1864)

59F9CDA8-596B-5293-B1F4-B134097D301F

##### Distribution

Cosmopolitan

##### Notes

S, e

**Reference**: Mock (2006)

#### Cylindroiulus latestriatus

(Curtis, 1845)

015DCC44-B303-577D-8F68-8D8FD23D8EBD

##### Distribution

Atlantic, North-western and Central European

##### Notes

S, e

**Reference**: Mock (2001b)

#### Cylindroiulus parisiorum

(Brölemann & Verhoeff, 1896)

2169FE95-512D-5B5C-9687-EE993EA34BAF

##### Materials

- **Type status:**Other material. **Occurrence:** individualCount: 21; sex: male; lifeStage: adult; **Taxon:** scientificName: *Cylindroiulusparisiorum* (Brölemann & Verhoeff, 1896); class: Diplopoda; family: Julidae; genus: Cylindroiulus; specificEpithet: *parisiorum*; taxonRank: species; **Location:** country: Slovakia; locality: Košice Basin, Košice – a city park (Mestský park) at the railway station; verbatimElevation: 210 m a.s.l.; locationRemarks: leaf litter (*Acer platanoides, Aesculus hippocastaneum*); verbatimCoordinates: 48°43'20.4"N 21°15'52.5"E; **Identification:** identifiedBy: A. Mock; **Event:** eventDate: 2018-12-18; fieldNotes: co-existing with *Nopoiuluskochii, Brachiulus lusitanus, Ophyiuluspilosus*; **Record Level:** basisOfRecord: PreservedSpecimen
- **Type status:**Other material. **Occurrence:** individualCount: 28; sex: female; lifeStage: adult; **Taxon:** scientificName: *Cylindroiulusparisiorum* (Brölemann & Verhoeff, 1896); class: Diplopoda; family: Julidae; genus: Cylindroiulus; specificEpithet: *parisiorum*; taxonRank: species; **Location:** country: Slovakia; locality: Košice Basin, Košice – a city park (Mestský park) at the railway station; verbatimElevation: 210 m a.s.l.; locationRemarks: leaf litter (*Acer platanoides, Aesculus hippocastaneum*); verbatimCoordinates: 48°43'20.4"N 21°15'52.5"E; **Identification:** identifiedBy: A. Mock; **Event:** eventDate: 2018-12-18; fieldNotes: co-existing with *Nopoiuluskochii, Brachiulus lusitanus, Ophyiuluspilosus*; **Record Level:** basisOfRecord: PreservedSpecimen
- **Type status:**Other material. **Occurrence:** individualCount: 28; sex: female; lifeStage: adult; **Taxon:** scientificName: *Cylindroiulusparisiorum* (Brölemann & Verhoeff, 1896); class: Diplopoda; family: Julidae; genus: Cylindroiulus; specificEpithet: *parisiorum*; taxonRank: species; **Location:** country: Slovakia; locality: Košice Basin, Košice – a city park (Mestský park) at the railway station; verbatimElevation: 210 m a.s.l.; locationRemarks: leaf litter (*Acer platanoides, Aesculus hippocastaneum*); verbatimCoordinates: 48°43'20.4"N 21°15'52.5"E; **Identification:** identifiedBy: A. Mock; **Event:** eventDate: 2018-12-18; fieldNotes: co-existing with *Nopoiuluskochii, Brachiulus lusitanus, Ophyiuluspilosus*; **Record Level:** basisOfRecord: PreservedSpecimen

##### Distribution

European

##### Notes

S, e, c

The species has European distribution; however, the captures apart from England are scattered and isolated. Its occurrence is associated with human activity. According to Kime and Enghoff (2017), it is relatively rare, some records are likely to be incorrect due to possible confusion with other *Cylindroiulus* species. Findings in synanthropic habitat represent the first records for Slovakia.

#### Cylindroiulus vulnerarius

(Berlese, 1888)

3F2C90F6-E45B-5522-BEE0-7729C555013F

##### Distribution

West and Central European

##### Notes

X, e, h

**Reference**: Mock (2001b)

#### Enantiulus nanus

(Latzel, 1884)

6A9BDF51-A050-5A30-8414-E0B0559A7BD7

##### Distribution

European

##### Notes

A, e, h

#### Enantiulus tatranus

(Verhoeff, 1907)

A4829F1E-DA65-527C-B7B1-D37F201BA8C6

##### Distribution

West Carpathian

##### Notes

R, e, m

#### Enantiulus transsilvanicus

(Verhoeff, 1899)

FC523E99-BEB5-5322-BB58-4FFED80B2107

##### Distribution

East Carpathian

##### Notes

R, e

**Reference**: Gulička et al. (2014)

#### Julus curvicornis

Verhoeff, 1899

C4854698-B0A2-5FEA-88FC-10B1D892E657

##### Distribution

Carpathian

##### Notes

R, e

#### Julus scandinavius

Latzel, 1884

838E1EA4-4893-559E-AC0B-12AF67C88986

##### Distribution

Central European and Scandinavian

##### Notes

A, e, n

#### Julus scanicus

Lohmander, 1925

ACE7AF66-74D7-5242-A32D-D26CEF6D988E

##### Distribution

Central European

##### Notes

A, e, h

#### Julus terrestris

Linnaeus, 1758

991BA89E-2910-5CC7-A987-3ADC15B2DB20

##### Distribution

North, Central, South and East European

##### Notes

R, e, h

#### Kryphioiulus occultus

(C. L. Koch, 1847)

F1365525-3B19-500B-BE44-03B9C771BE26

##### Distribution

East European

##### Notes

A, e, x

#### Leptoiulus baconyensis

(Verhoeff, 1899)

15D295F6-7BF1-5386-A52E-66E7A0C596D2

##### Distribution

East and Central European

##### Notes

R, e

#### Leptoiulus cibdellus

(Chamberlin, 1921)

08E79850-A0D8-5202-90FB-1DCFEE256CFF

##### Distribution

North and Central European, Baltic

##### Notes

R, e, h, n

#### Leptoiulus liptauensis

(Verhoeff, 1899)

37631AAA-BF6F-5888-9930-B3D690E6A46D

##### Distribution

West Carpathian

##### Notes

R, e, m

#### Leptoiulus noricus

Verhoeff, 1913

8C2CE56F-DB40-5F25-A980-976BB5F8386E

##### Distribution

Sudetico-Carpathian

##### Notes

R, e, m

#### Leptoiulus mariae

Gulička, 1952

528708CF-BDA4-5421-8648-F5613BD4F96A

##### Distribution

West Carpathian

##### Notes

R, e, tp

#### Leptoiulus proximus

(Němec, 1896)

5D4CD2CC-5E74-584E-B453-8BD294C5FA8D

##### Distribution

North, Central and East European

##### Notes

A, e

#### Leptoiulus tatricus

Gulička, 1956

5CFD59DC-D3C9-51A1-A7EC-982F005C6A4B

##### Distribution

West Carpathian

##### Notes

R, e, m

#### Leptoiulus trilobatus

(Verhoeff, 1894)

E70CBA85-A434-5CCF-8054-9F30CB9DE647

##### Distribution

Central and East European

##### Notes

A, e

#### Leptoiulus tussilaginis

(Verhoeff, 1907)

10EE350B-3B20-5539-A3F8-F06E5320DA26

##### Distribution

West Carpathian

##### Notes

R, e, m

#### Megaphyllum projectum

Verhoeff, 1894

2F4ADCC2-0CAE-5197-96DE-987A6C1EE653

##### Distribution

Central and East European

##### Notes

A, e

#### Megaphyllum silvaticum

(Verhoeff, 1898)

18575707-9CD0-58B8-AC98-F6C577D56B10

##### Distribution

East and Central European

##### Notes

R, e, m

#### Megaphyllum unilineatum

(C. L. Koch, 1838)

82675623-7D8A-587F-9DA2-ECC464C45BD1

##### Distribution

South and Central European, Balkan

##### Notes

E, e, x

#### Ommatoiulus sabulosus

(Linnaeus, 1758)

F3581CAB-3DDD-5006-9F26-42C2AB926E38

##### Distribution

European

##### Notes

E, e, x

#### Ophyiulus pilosus

(Newport, 1842)

002768BF-03DC-5C20-AF68-E1D1D6813B89

##### Distribution

European

##### Notes

E, e

#### Unciger foetidus

(C. L. Koch, 1838)

968A4371-833F-5CF1-8DEB-6403DC30EB83

##### Distribution

European

##### Notes

E, e, h

#### Unciger transsilvanicus

(Verhoeff, 1899)

590A2C07-04D4-5B08-82B9-6E7AB7E2C297

##### Distribution

Central European

##### Notes

A, e

#### Xestoiulus carpathicus

(Verhoeff, 1907)

2AC4FCC1-001A-5F77-88D6-0BF22F3560D6

##### Distribution

North Carpathian

##### Notes

R, e, m

#### Xestoiulus laeticollis

(Porat, 1889)

9DECA411-8B99-5EBF-BFCC-9236A24D3D0B

##### Distribution

East and Central European

##### Notes

R, e, h, n

**Reference**: Tajovský et al. (2001)

#### Chordeumatida

0E3D0A19-D96F-53D6-B9B2-04BDD47C8879

#### Chordeumatidae

F11EB5B5-46CA-5729-AA43-7CDCC80AFBAA

#### Melogona broelemanni

(Verhoeff, 1897)

8779CE31-7CCF-5ED2-844B-F748C93AF4F1

##### Distribution

Central European, Balkan

##### Notes

S, e

**Reference**: Mock and Tajovský (2002)

#### Melogona transsylvanica

(Verhoeff, 1897)

160BA32D-8C74-58FD-A7F6-B98739996D13

##### Distribution

East Carpathian

##### Notes

A, e

**Reference**: Mock and Tajovský (2002)

#### Melogona voigtii

(Verhoeff, 1899)

A36ACD63-2589-50F5-BCEE-180E889D5584

##### Distribution

North and Central European

##### Notes

S, e

**Reference**: Mock and Tajovský (2002)

#### Brachychaeteumatidae

331205F4-1713-5FBA-8F29-22CB684628EA

#### Brachychaeteuma bradeae

(Brolemann & Brade-Birks, 1917)

CD723F79-E5EA-5909-A6A2-ABF03F0F0BAC

##### Distribution

West European

##### Notes

R, tp

**Reference**: Kováč et al. (2005)

#### Trachygonidae

BCF46DAF-6878-5C36-8C31-E0AFFA337A96

#### Heteroacrochordum evae

Loksa, 1960

FF9DAFFD-2F98-5440-B74F-938C13BBFF45

##### Distribution

West Carpathian

##### Notes

R, ed

**Reference**: Mock et al. (2019)

#### Hungarosomatidae

E6E1CFB1-CB6A-50D7-BF31-8C5C8BFE1358

#### Hungarosoma bokori

Verhoeff, 1928

10E8DB47-2C13-54BF-97D3-62954C7C3AB6

##### Distribution

West Carpathian

##### Notes

R, e, h

The first description of the species was published by Verhoeff (1928), based on a single female specimen from the Abaliget Cave in Hungary. Detailed analysis of diagnostic characteristics, based on the fresh material from the type locality, as well as all available museum material, was presented only recently by Mock et al. (2016). However, the authors overlooked the apparent similarity of the diagnostic features with that of *Ochogonacervina* (Verhoeff, 1899), recently pointed out by Antić et al. (2018). Gonopods of both species appear to be identical, nevertheless, a synonymy was not formally established. Minute differences between Verhoeff’s description of *O.cervina* and our knowledge of *H.bokori* must be reviewed. In addition, in order to justify the name of the family Hungarosomatidae and its position in the Chordeumatida system, the decision as to which genus the species belongs: *Ochogona*, *Octeicosisoma*, *Triakontizona* or *Ceratosoma*, has to be resolved (Haľková and Mock 2018, Korsós and Lazányi 2020).

**Reference**: Papáč et al. (2014)

#### Craspedosomatidae

0A4EF9DD-BE59-578D-AE90-9AF360667BBF

#### Beskidia jankowskii

(Jawlowski, 1938)

5465705D-4E49-593D-8E26-4B084410442C

##### Distribution

East Carpathian

##### Notes

R, e, m

**Reference**: Gulička et al. (2014)

#### Craspedosoma raulinsii

Leach, 1814

18001744-0F61-565E-9304-C5DC381DEE11

##### Distribution

West, Central and South European

##### Notes

A, e

#### Craspedosoma transsilvanicum

Verhoeff, 1897

C034B994-8521-5B2A-BF62-86B943A61F9C

##### Distribution

South and Eastern European

##### Notes

R, e

#### Chelogona carpathicum

(Latzel, 1882)

9791AA65-998E-5E80-8ACA-379C1C6853A6

##### Distribution

West Carpathian

##### Notes

R, e, m

#### Ochogona caroli

(Rothenbuhler, 1900)

F85EE3C3-6D8D-598A-800C-A0E921089325

##### Distribution

Central European

##### Notes

R, e

**Reference**: Gulička et al. (2014)

#### Haasidae

8465A86A-22AF-58EA-A29D-62CCEF45F63C

#### Haasea flavescens

(Latzel, 1884)

C84E98DF-B346-5CFA-997A-1DBA43A3F8F5

##### Distribution

South and Central European

##### Notes

A, e

#### Hylebainosoma gulickai

(Tajovský, Mock & Papáč, 2014)

A999896F-BD64-58F6-A12B-B3C7541A1B9F

##### Distribution

West Carpathian

##### Notes

R, tb

#### Hylebainosoma tatranum

Verhoeff, 1899

140D933F-8B82-55A7-98C8-2C3B87E733AD

##### Distribution

North Carpathian

##### Notes

R, e, m

#### Entomobielziidae

E7D5C923-C05B-5F7F-9308-8679655690C8

#### Entomobielzia kimakowizii

(Verhoeff, 1897)

A238419E-E84D-5864-94E8-30FCFEB8B7DA

##### Distribution

East Carpathian

##### Notes

R, e

**Reference**: Gulička et al. (2014)

#### Attemsiidae

65C7370E-1D2E-5072-9B36-54FF3C108D9C

#### Allorhiscosoma sphinx

(Verhoeff, 1907)

2A92FCE4-9022-5340-833A-B55860611281

##### Distribution

West Carpathian

##### Notes

R, tp

#### Mecogonopodium carpathicum

Mock & Tajovský, 2008

8E374C6F-3304-51BB-B71F-3023B348134D

##### Distribution

West Carpathian

##### Notes

R, tp

#### Mastigophorophyllidae

F3341C42-CE6E-5A79-97D3-5A0080CB7F45

#### Haploporatia eremita

(Verhoeff, 1909)

9CADC961-AE1B-5FD5-9F57-4B13B5EA3CCF

##### Distribution

Central European

##### Notes

R, e

#### Mastigona bosniensis

(Verhoeff, 1897)

10F158A8-343B-5272-98A4-A5C356723B03

##### Distribution

Central and East European

##### Notes

E, e

#### Mastigophorophyllon cirriferum

Verhoeff, 1899

304B8977-AF62-58F9-BDF5-7CCD99B24798

##### Distribution

West Carpathian

##### Notes

R, e, m

#### Verhoeffiidae

725C200A-8CB2-5B3F-899B-73FEFDE65557

#### Haplogona oculodistincta

(Verhoeff, 1893)

3B6E25C9-4384-59EA-8A19-7E147787968D

##### Distribution

Alpine

##### Notes

S, e

**Reference**: Haľková and Mock (2018)

#### Polydesmida

04B3F154-EEB0-589A-AAB6-EA53CFF83B6F

#### Polydesmidae

CE25DD76-98C6-5791-BFB1-E37E5C39CA55

#### Brachydesmus dadayi

Verhoeff, 1895

533BBE4D-9FCB-563B-B251-E8258224F4C2

##### Distribution

South, Central and East European

##### Notes

A, e, h

#### Brachydesmus superus

Latzel, 1884

694DE3E8-C9E8-52CC-A576-03D8521719E2

##### Distribution

European

##### Notes

S, e, h

#### Polydesmus burzenlandicus

Verhoeff, 1925

3F6D92C3-5485-51AF-A747-5E482E314050

##### Distribution

South-eastern Carpathian

##### Notes

R, e, c

*Polydesmusburzenlandicus* is a small hygrophilous representative of the order Polydesmida, widespread mainly in the south-eastern Carpathians (Kime and Enghoff 2011) The species inhabits forest habitats of the mountainous landscape. It was recently documented from the area of the Latorica PLA (Mock et al. 2021). These findings represent the first record for Slovakia.

**Reference**: Mock et al. (2021)

#### Polydesmus complanatus

(Linnaeus, 1761)

2F776C36-2364-557E-9339-FA5E7F1A6C08

##### Distribution

North, Central, South and East European

##### Notes

E, e, h

#### Polydesmus denticulatus

C. L. Koch, 1847

DFBCDACE-498E-578F-9B2B-6666B24578C4

##### Distribution

European

##### Notes

E, e

#### Polydesmus inconstans

Latzel, 1884

41C36D84-A0C7-5A99-A3F6-40C3FF568B9B

##### Distribution

European

##### Notes

S, e, h

**Reference**: Mock (2004)

#### Polydesmus komareki

Ložek & Gulička, 1962

7A252CA8-152E-5F89-9304-7D099E79DD1B

##### Distribution

East Carpathian

##### Notes

R, e, m

#### Polydesmus montanus

Daday, 1889

AED868E6-77C3-5328-A02B-3A599AD52E30

##### Distribution

East Carpathian

##### Notes

R, e, c, m

#### Polydesmus polonicus

Latzel, 1884

7602842C-F4AB-568C-A312-4A9572FEE2B7

##### Distribution

East Carpathian

##### Notes

R, e, m

#### Polydesmus subscabratus

Latzel, 1884

8387BDA8-3A08-5193-B8A5-708A271BC0AB

##### Distribution

South Carpathian

##### Notes

R, e, tp, ?

South Carpathian endemic species with affinity to wet forested habitats. From Slovakia, only one questionable historical record is documented from the vicinity of the Veľaty Village (Daday 1889), significantly isolated from the rest of the area of its known distribution in the Southern Carpathians (Kime and Enghoff 2011, Kime and Enghoff 2013). Its occurrence in Slovakia, however, can be supported by the recent findings of *P.transylvanicus* Daday, 1889 in nearby locations within the Eastern Slovak Plain (Haľková and Mock 2018), the species with similar biogeography and ecology as *P.subscabratus*.

#### Polydesmus tatranus

Latzel, 1884

6135C067-D8A4-5AEF-B37B-BDACE0170229

##### Distribution

Carpathian

##### Notes

R, e, h, m

#### Polydesmus transylvanicus

Daday, 1889

F19BF5DF-6F23-5EBD-BC58-0DDB68335FC0

##### Distribution

Carpathian

##### Notes

R, e, h

**Reference**: Haľková and Mock (2018)

#### Paradoxosomatidae

CA7A8050-3949-55B0-8481-75FBA30951DF

#### Oxidus gracilis

(C. L. Koch, 1847)

00AF0722-FD46-50DD-A656-DB0CEE6CB79E

##### Distribution

Cosmopolitan

##### Notes

X, e

#### Strongylosoma stigmatosum

(Eichwald, 1830)

58E29EC2-4A4D-5FF3-8A8E-6095FD2EB639

##### Distribution

East and Central European, Baltic

##### Notes

A, e, tp, h

#### Pyrgodesmidae

39B0E373-F2B2-5294-A509-D0FC38803166

#### Poratia digitata

(Porat, 1889)

8C690F2D-6B47-56FC-B104-FF46490EB680

##### Distribution

North, West and Central European

##### Notes

X, e, c, h

**Reference**: Mock (2001b)

#### Oniscodesmidae

5A793D16-C55A-5C43-9D9B-5CC8B6D5FECA

#### Amphitomeus attemsi

(Schubart, 1934)

FC24AFCE-C982-58AC-A70A-8C2F9A09C482

##### Distribution

East and Central European

##### Notes

X, e, c, h

**Reference**: Mock (2001b)

## Discussion

The increase in faunistic research over the past two decades has resulted in significant expansion of knowledge of the Slovak millipede fauna. Since the last published review of millipede species from the territory of Slovakia (Mock 2001a), 23 species have been added, resulting at present in 93 species. Within the whole Carpathian Region, Romania still represents the country with the richest fauna, with 170 recorded millipede species (Giurginca 2021). Nevertheless, compared to the total species number of millipedes from neighbouring countries, 94 from Poland (Stojałowska and Staręga 1974, Wytwer 1997), 77 from the Czech Republic (Tajovský and Tuf 2016, Kocourek et al. 2017), 107 species from Hungary (Korsós and Lazányi 2020), 190 from Austria (extrapolation, Geiser 2018) and 75 from Germany (Hauser and Voigtländer 2019), 93 species recorded for Slovakia constitute a strikingly high number, considering the rather small area of the country. In addition, new findings are still expected for the whole territory of Slovakia since numerous locations remain less investigated, as well as records of non-native species are likely to emerge.

Most of the unclear and questionable data that were preliminarily excluded from the list are from the older literature. One of the most recent studies, creating confusion due to imprecision in accurate and reliable determination, was published by Topp et al. (2006). The authors investigated the biodiversity of woodlice and millipede faunas of the primeval forest of Central Slovakia. The authors' team, however, lacked a taxonomist and, as a result, species not occurring in our country or species with strictly synanthropic occurrence were listed, such as such as *Mycogonagermanica*, *Allaiulusnitidus* or *Blaniulusguttulatus* and *Cylindroiuluslatestriatus* (as *Aneuloboiulusfrisius*). From the faunistic point of view, the article is very inaccurate and should be approached with great caution.

The millipede fauna of Slovakia can be characterised as a combination of European, Alpine-Atlantic and Carpathian elements, with the occasional influence of Caucasian and Balkan fauna (Kime and Enghoff 2011, Kime and Enghoff 2017). From the biogeographical aspect, a relatively high number of endemic species are represented in the country. This is presumably linked to the Carpathians. Compared to the Alps, the geographical repartition of the Carpathian fauna was less affected by glaciations, allowing the isolation of the Carpathian Regions surrounded by the Paratethys (Holdhaus 1954). The presence of a significant proportion of endemic fauna characterises the millipede fauna of Slovakia as exceptional in Central Europe. Gulička and Košel (2016) have mentioned the presence of 28 Carpathian endemic species of millipedes in Slovakia. A large part of these species is defined as Western-Carpathian. However, the border between the Eastern and Western Carpathians crosses the eastern part of the country, resulting in the representation of the eastern Carpathian fauna. Nevertheless, it should be noted that this border is not strict and is not consistent for all the species. For some species, the term Northern-Carpathian endemic is more appropriate. Some species even can be characterised by microendemism (e.g. *Leptoiulustatricus*, *Mecogonopodiumcarpathicum* and *Hylebainosomagulickai*). The presence of the Western Carpathians has an undeniable influence on the composition of Slovak millipedes. Almost 20% of the species are represented by rare mountainous fauna and more than 10% are characteristic for their preference of cave habitat.

According to ecological classification, 50% of Slovak millipede fauna are represented by stenotopic species inhabiting exclusively undisturbed habitats with a low impact of human activities. A total of 20% can be classified as adaptable and only 7% can be classified as eurytopic. While the original classification, proposed by Tuf and Tufová (2008), is applicable to most of the species, the categorisation does not sufficiently segregate non-native, introduced species occurring exclusively in a specific environment (e.g. tropical species in greenhouses). Therefore, we added and used two categories to this classification, in order to distinguish synanthropic and exotic species. These categories include the remaining 13% of millipede species of Slovakia.

Despite the increased interest in faunistic research, the millipede fauna of Slovakia has not yet been completely investigated. Several findings are still to be determined at the species level. Such findings include subadult and female individuals of *Typhloiulus* sp. from the Domica Cave (Papáč et al. 2014) and a representative of the family Trichopolydesmidae (Akkari and Enghoff 2011). Both taxa are connected to the cave environment.
